# Draft genome sequences of *Escherichia* spp. isolates from New Zealand environmental sources

**DOI:** 10.1128/mra.01007-23

**Published:** 2024-02-20

**Authors:** Patrick J. Biggs, Marie Moinet, Lynn E. Rogers, Megan Devane, Richard Muirhead, Rebecca Stott, Jonathann C. Marshall, Adrian L. Cookson

**Affiliations:** 1 mEpiLab, Hopkirk Research Institute, School of Veterinary Science, Massey University, Palmerston North, New Zealand; 2 School of Natural Sciences, Massey University, Palmerston North, New Zealand; 3 New Zealand Food Science and Safety Research Centre, Massey University, Palmerston North, New Zealand; 4 AgResearch, Hopkirk Research Institute, Palmerston North, New Zealand; 5 Water and Health, Institute of Environmental Science and Research Ltd. (ESR), Christchurch, New Zealand; 6 AgResearch, Invermay Agricultural Centre, Mosgiel, New Zealand; 7 Environmental Health, National Institute of Water and Atmospheric Research (NIWA), Hamilton, New Zealand; 8 School of Mathematical and Computational Sciences, Massey University, Palmerston North, New Zealand; Queens College Department of Biology, USA

**Keywords:** *Escherichia* spp., water quality, *Escherichia marmotae*, *Escherichia whittamii*, *Escherichia ruysiae*

## Abstract

*Escherichia coli* is often used as a fecal indicator bacterium for water quality monitoring. We report the draft genome sequences of 500 *Escherichia* isolates including newly described *Escherichia* species, namely *Escherichia marmotae*, *Escherichia ruysiae,* and *Escherichia whittamii*, obtained from diverse environmental sources to assist with improved public health risk assessments.

## ANNOUNCEMENT

Naturally occurring *Escherichia coli* from the gut of warm-blooded animals (including birds) is a common indicator of fecal contamination for freshwater quality monitoring as a proxy for fecal contamination and pathogens (1). However, current culture-based methods, used to enumerate *E. coli*, cannot distinguish between fecal *E. coli* and naturalized or environmental associated “*E. coli*-like” strains, also known as *Escherichia* Cryptic Clades (2–4). *Escherichia whittamii* (Cryptic Clade 2) (5), *Escherichia ruysiae* (Cryptic Clades 3 and 4) (6), and *Escherichia marmotae* (Cryptic Clade 5) (7) are recently described taxa, but host species and environmental persistence remain to be established. This project focuses on the whole-genome sequencing of *E. coli* and *Escherichia* spp. from environmental sources (freshwater, riverine sediment, aquatic biofilm, soil and fecal material from birds, and mammals). Strains were obtained as part of a study examining the impact of contrasting land uses on *Escherichia* spp. and were cultured as described previously (8). Genomic data from *E. coli* and the new *Escherichia* spp. will provide information on the environmental survival of these bacteria and more accurate fecal tracking, enabling the most impactful sources of contamination affecting waterways to be identified and rapidly addressed.

Each strain was resuscitated from freezer stocks and grown on sheep blood agar (Fort Richard, New Zealand) at 37°C. A single colony was subcultured onto fresh sheep blood agar, with DNA extractions undertaken from five colonies for each strain using the Wizard Genomic DNA Purification Kit (Promega, WI, USA). Sequencing libraries were prepared using the Nextera XT DNA library preparation kit (Illumina, CA, USA) and sequenced on either the Illumina MiSeq or HiSeq X platform. The resultant 2 × 150 base paired-end reads were quality assessed with FastQC (v0.11.9) (9). *De novo*-assembled genomes were obtained using the Nullarbor pipeline (2.0.20191013) (10), including Trimmomatic (v0.39) (11), and assembled with SKESA (v2.4.0) (12) using the respective default parameters for each tool. The genomes were automatically annotated using the NCBI Prokaryotic Genome Annotation Pipeline (13).

Average depth coverage for the draft genomes was 101× (range 28–437×), with average genome size of 4,904,079 bp (range 4,287,000–5,664,657 bp), average number of contigs 107 (range 26–356), and average *N*_50_ of 145,778 bp (range 38,436–716,300 bp). The genomes were categorized according to source (Table 1) with those from feces (*n* = 182) the most common. Mash-based phylogroups were assigned *in silico* from assembled genomes using the ClermonTyper web tool (14). Eight established *E. coli* phylogroups were identified: A (*n* = 6), B1 (*n* = 182), B2 (*n* = 80), C (*n* = 8), D (*n* = 56), E (*n* = 15), F (*n* = 4), and G (*n* = 1), as well as Cryptic Clade 1 (*n* = 1) (15, 16). In addition, 147 isolates were identified as non-*E*. *coli Escherichia* species: *Escherichia whittamii* (Cryptic Clade 2, *n* = 2), *Escherichia ruysiae* (Cryptic Clade 3, *n* = 4), and *Escherichia marmotae* (Cryptic Clade 5, *n* = 141).

**TABLE 1:** Summary of 500 genomes from environmental sources

Source	No. of genomes	Phylogroups observed	Cryptic lineages observed
Feces (avian)	166	B1, B2, C, D, E, F	2, 3, 5
Feces (mammal)	14	B1, C, D	None
Feces (unknown)	2	B1	5
Biofilm	90	B1, B2, C, D, E, G	1, 5
Sediment	78	A, B1, B2, D, E	5
Soil	45	A, B1, B2, D, E	5
Water	105	A, B1, B2, C, D, F	5

## Data Availability

The draft genome assemblies (JAVGZV010000000–JAVHJK010000000) and short reads were deposited at DDBJ/ENA/GenBank under BioProject accession number PRJNA1007421. A full listing of the source and phylogroup information for the 500 genomes can be found at https://doi.org/10.6084/m9.figshare.24152268 (17).
